# Noninvasive Monitoring of Glucose Using Near-Infrared Reflection Spectroscopy of Skin—Constraints and Effective Novel Strategy in Multivariate Calibration

**DOI:** 10.3390/bios11030064

**Published:** 2021-02-27

**Authors:** H. Michael Heise, Sven Delbeck, Ralf Marbach

**Affiliations:** 1 Interdisciplinary Center for Life Sciences, South-Westphalia University of Applied Sciences, Frauenstuhlweg 31, 58644 Iserlohn, Germany; Delbeck.Sven@fh-swf.de; 2 CLAAS Selbstfahrende Erntemaschinen, Muehlenwinkel 1, 33428 Harsewinkel, Germany; Ralf.Marbach@claas.com

**Keywords:** noninvasive glucose sensing, near-infrared spectroscopy, skin tissue reflection spectroscopy, calibration modeling, science-based calibration (SBC)

## Abstract

For many years, successful noninvasive blood glucose monitoring assays have been announced, among which near-infrared (NIR) spectroscopy of skin is a promising analytical method. Owing to the tiny absorption bands of the glucose buried among a dominating variable spectral background, multivariate calibration is required to achieve applicability for blood glucose self-monitoring. The most useful spectral range with important analyte fingerprint signatures is the NIR spectral interval containing combination and overtone vibration band regions. A strategy called science-based calibration (SBC) has been developed that relies on a priori information of the glucose signal (“response spectrum”) and the spectral noise, i.e., estimates of the variance of a sample population with negligible glucose dynamics. For the SBC method using transcutaneous reflection skin spectra, the response spectrum requires scaling due to the wavelength-dependent photon penetration depth, as obtained by Monte Carlo simulations of photon migration based on estimates of optical tissue constants. Results for tissue glucose concentrations are presented using lip NIR-spectra of a type-1 diabetic subject recorded under modified oral glucose tolerance test (OGTT) conditions. The results from the SBC method are extremely promising, as statistical calibrations show limitations under the conditions of ill-posed equation systems as experienced for tissue measurements. The temporal profile differences between the glucose concentration in blood and skin tissue were discussed in detail but needed to be further evaluated.

## 1. Introduction

The advantages of tight glycemic control in people with diabetes mellitus have often been documented since the diabetes control and complications trial (DCCT) studies were completed [1,2,3]. Those studies proved that intensive insulin therapy in such patients could dramatically delay many serious complications caused by an increase of glycation of body proteins due to above-average blood glucose concentration, which can also be connected to problems from micro- and macrovascular diseases leading, e.g., to retino-, nephro-, and neuropathy. Most diabetic patients use the equipment for blood glucose self-monitoring (SMBG) that tracks their glucose concentrations and enables them to adjust their insulin dosage and achieve normoglycemia. Over the past few years, substantial progress can be seen in research to find improved devices for diabetic patients, mostly based on electrochemical and optical sensors; for an overview on current methods and instrumentation, see recent reviews [4,5,6,7,8,9,10,11]. When undergoing intensive insulin therapy, current surveillance still requires people with diabetes to use lancets to prick their fingertips for blood sampling several times a day. Alternatively, they can use continuous glucose monitoring (CGM) devices that have recently been brought to the market. As so-called non-adjunctive devices, these still require invasive blood testing from time-to-time. Such factory-calibrated sensors are used for intermittently scanned continuous glucose monitoring but still face limitations. Problems may occur in situations with rapid blood glucose changes [12,13], and sensor glue can cause skin irritations [14]. Despite this, minimal-invasive continuous glucose sensing systems have been suggested for glycemic control in people with diabetes mellitus and critically ill patients [15].

A noninvasive measurement system certainly eliminates the inconvenience and pain of multiple daily blood tests and, as observed with continuously monitoring devices, avoids the invasiveness of today’s flash glucose monitoring sensors or microdialysis catheter implants combined with ex vivo detection. Noninvasive instrumentation also allows a larger number of measurements than using SMBG invasive devices.

A multitude of optical methods has been suggested for the development of noninvasive methods of blood glucose monitoring. To date, applied spectroscopic methods have been based on vibrational spectroscopy and include mid-infrared, NIR and Raman spectroscopy, among other techniques such as fluorescence, polarimetry, and optical coherence tomography, for which recent comprehensive reviews exist, e.g., [8,9,10,11]. New publications based on mid-infrared [16] or Raman spectroscopy [17] have shown promising results for achieving noninvasive assays, and earlier papers from both research teams provide more insight into their measurement techniques [18,19].

For many years, NIR spectroscopy has found application in clinical chemistry. In particular, glucose quantification in serum, plasma, or whole blood has been demonstrated successfully by several authors; see, for example, [20,21,22,23]. Therefore, several projects were started for the development of noninvasive assays based on skin measurements. An interesting and fascinating book to read on the many fruitless efforts in the past has been published by Smith [24]. Despite those failures, NIR spectroscopy offers a substantial potential for medical applications, including noninvasive methodology for blood glucose determinations; for an overview, see a recent book chapter [25]. Skin spectroscopy based on transmission measurements requires thin skin folds or short-wave NIR spectroscopy for transilluminating a fingertip or an earlobe [26]. For accessing information-rich spectral intervals with first overtone and combination band vibrations, the diffuse reflection technique has been favored when measuring skin.

The noninvasive sensing of glucose is experimentally challenging due to the tiny glucose absorbance, a dominating high and variable background absorption of water, baseline shifts, instrumental drift, lack of sensitivity, and poor precision. Multivariate calibrations are required to allow for the obligatory selectivity [27] of reliable glucose quantification with large-enough analyte absorbances above the noise level. Traditionally, two different calibration modeling approaches are used. Analytical spectroscopists were analyzing sample spectra by least-squares fitting with reference absorptivity spectra of analytes contributing—in most cases—linearly to the sample spectrum, dependent on their concentrations. The physics behind this is the validity of Beer’s law. This approach for glucose sensing is known as “classical least squares” (CLS) calibration [28] and was suggested by Maruo and Yamada [29] under the assumption that absorbance difference spectra of human forearm skin versus that of a start spectrum can be modeled by a linear combination of spectra of glucose, water, protein, fat, and a baseline for scattering.

The other more widely applied modeling technique relies on statistical calibration (also called inverse calibration) based on traditional partial least-squares (PLS), principal component regression (PCR), or, more recently, machine learning tools; for recently published examples of use in noninvasive methodology testing, see Refs [16,17]. The reader is also referred to our earlier publications for insight into previously favored calibration modeling and its advantages and disadvantages [28,30]. For proving the required selectivity, the net analyte signal (NAS) has been suggested as an approach to validate the spectrometric model when separating the glucose spectral signature from those of the tissue matrix components [31,32].

Most projects for the development of noninvasive glucose assays, including our own work, used statistical PLS calibrations with sophisticated data treatment and variable selection methods [33,34,35,36]. It could also be demonstrated that problems and pitfalls can arise from overfitting due to the implementation of too many spectral variables or insufficient model validation [33]. If a statistical calibration technique is used, there are additional problems such as a nonspecific response or an implementation of spuriously correlated spectral variance into the calibration model. Further evaluations of such spectrometric assays, including a discussion of problems and perspectives, have been published in the past [31,33].

Another approach, originally called “spectral Wiener filtering” and known from time signal processing theory, has been developed and successfully tested. The results of this approach are presented here, combining a priori information such as the spectral absorptivities of the analyte of interest with estimates of the variance of the population with negligible analyte concentration dynamics [37,38,39]. When compared to calibration modeling based on PLS and with regard to workload, this method is also less expensive allows, without an analytical reference method, the specificity of response to be proven from first principles, and combines the best features of both worlds, i.e., of the physical and statistical modeling approaches. Early users from the pharmaceutical industry, working with process analytical technologies (PAT), referred to this as a “science-based” method, so the name “science-based calibration” (SBC) method was created. The calibration method has been implemented several times for pharmaceutical applications, such as in a tablet-coating process using Raman spectra [40].

In the context of clinical chemistry for glucose quantification, this method was successfully tested on the NIR-spectra of plasma samples obtained using a thermostated cell with a constant pathlength of 1 mm. Compared to previous PLS calibration models, the results were favorable [33]. For transcutaneous spectra obtained by diffuse reflection, estimating the glucose “response spectrum” is more difficult since the wavelength-dependent photon penetration depth into the skin requires a wavelength-dependent scaling of the aqueous glucose absorptivity spectrum. The scaling can be obtained from optical skin parameters such as absorption and scattering constants. Results for glucose concentration in the lip mucosa tissue of a diabetic subject, recorded under modified oral glucose tolerance test conditions, will be presented using scaling parameters as obtained for dermis and lip spectra. For the first time, results are shown for tissue glucose concentrations that differ, as expected, from blood glucose measurement as the current gold standard for diabetes therapy. Several publications addressed the time delay observed in measurements within the interstitial tissue compartment as accessible with invasive needle-type biosensors. However, the present results provide insight into integral tissue measurements with vascular, interstitial, and intracellular glucose-containing aqueous compartments.

## 2. Materials and Methods

### 2.1. Spectrometer Hardware and Recorded Spectra

**Figure 1:**
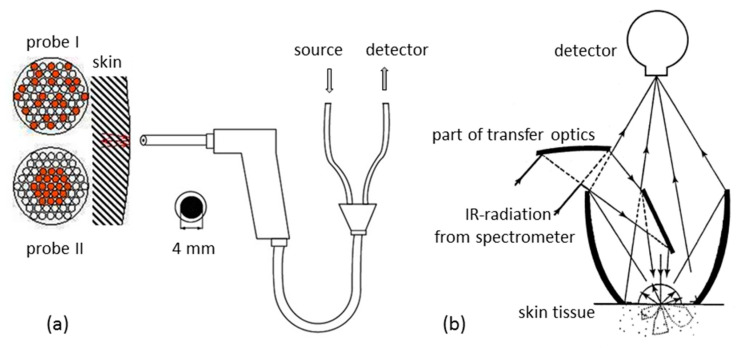
Diffuse reflection accessories used for skin measurements: (**a**) fiber-optic probes with fibers for illumination and detection arranged differently; (**b**) mirror-based device providing tissue spectra with different probing depths.

Within the so-called “therapeutic window”—as coined by biospectroscopists—with wavenumbers between 16,600 cm^−1^ and 7700 cm^−1^, a transmission measurement, e.g., of a fingertip, is feasible with tissue absorption small compared to scattering [26]. For shorter wavenumbers, such NIR-spectral measurements lead to extremely low transmittance values, whereas experiments using diffuse reflectance (DR) can easily be realized. Different accessories were employed based on either quartz fibers or mirror optics (see Figure 1). With fiber-optic probes, optimal distances between tissue illuminating fibers and detection fibers can be arranged to reach a certain skin depth and to probe the dermis vasculature as well (see also different fiber arrangements illustrated in Figure 1a). This approach made use of an optimized accessory with a rotational ellipsoidal mirror, which produced the highest signal-to-noise ratios, good reproducibility of the lip measurement, and allowed a temperature control of the lip contact area at 37 °C [41].

**Figure 2:**
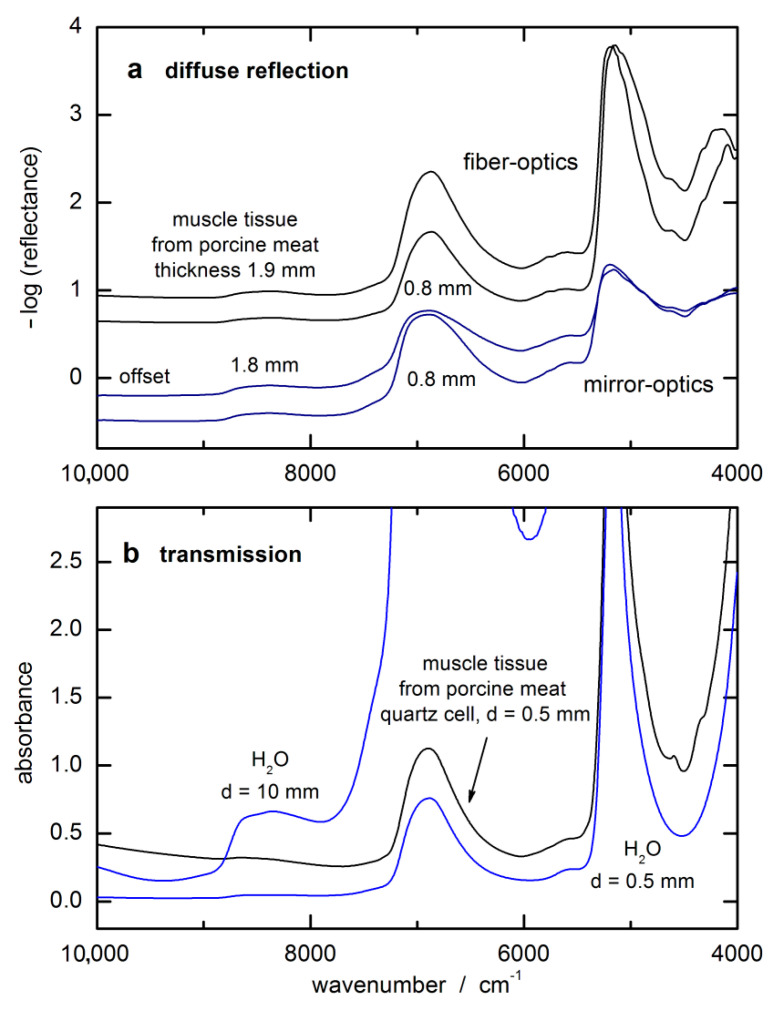
Spectra of tissue phantoms of constant layer thicknesses illustrating wavelength-dependent radiation penetration: (**a**) monitored with different accessories (for the first two spectra, a fiber-optic probe of type I was used); (**b**) transmission spectra of water and tissue.

In Figure 2a, examples of spectra of different layers of muscle tissue backed with a reflecting gold-coated carrier substrate are shown that were measured in diffuse reflection. It shows that the spectral intensities are accessory-dependent due to the accessible solid angle for detection of the back-scattered photons. For some spectral intervals, an enlargement of the tissue thickness does not lead to larger absorbance values, indicating that “saturation” was already reached at an even shorter penetration depth than the thinnest layer of 0.8 mm would allow. For comparison, an absorbance spectrum from a 0.5 mm thick tissue layer, enclosed by quartz windows and measured in transmission, is shown together with water spectra that were measured in cuvettes of different pathlengths (see Figure 2b). In comparison with absorbance spectra of liquid water, a transmission equivalent sample thickness can be estimated for the spectra measured by diffuse reflection spectroscopy.

Mucous lip tissue was chosen for the transcutaneous measurements because it is rich in capillary blood vessels (equivalent to an advantageously high blood volume inside the probed tissue) and also provides good optical contact to the constructed diffuse reflection accessory for recording high-quality in vivo spectra. NIR-spectra of the inner lip were recorded using a Fourier Transform spectrometer (model IFS-66 from Bruker Analytische Messtechnik, Ettlingen, Germany) and a liquid nitrogen-cooled InSb detector (Infrared Associates, Suffolk, UK). An on-axis ellipsoidal mirror is the essential optics part of the reflection accessory that collects the diffusely back-reflected radiation from the skin tissue in a very efficient manner and much differently from commercially available accessories or fiber-optic probes [41,42]. Reflectance spectra R(ῦ) = I_s_(ῦ)/I_0_(ῦ) were calculated with I_s_(ῦ) and I_0_(ῦ), i.e., the single-beam lip spectrum and the reflectance standard, respectively. Previously, also spectra of standards of different reflectivity were measured, but the one with a reflectivity of 5%, also from Spectralon (Labsphere, North Sutton, NH, USA), was preferred for matching the reference standard interferogram to that of the lip spectrum in an optimal manner by comparing their interferogram maxima.

**Figure 3:**
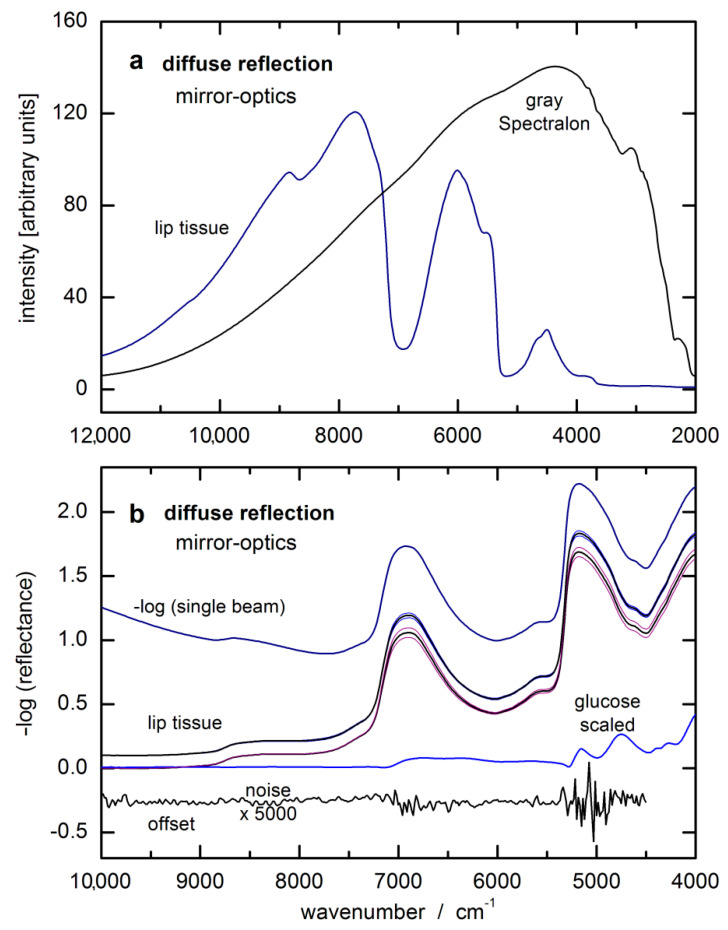
(**a**) Single-beam spectra of oral mucosa and a gray reflectance standard of 5% reflectivity, measured by diffuse reflection; (**b**) log-converted single-beam lip spectrum and two lip reflectance spectra as calculated from two respective series of five consecutively recorded lip tissue spectra shown as average spectra with according standard deviation spectra (spectra were taken at the beginning of our two-day measurements with capillary blood glucose values around 50 mg/dL and around 400 mg/dL during the first day; all spectra were offset for better visualization) and enlarged noise spectrum from two consecutive lip measurements after baseline correction.

Before the lip measurements were started, the test person had to rinse their mouth with plain water. After patting the lip dry, the spectra were taken reproducibly by slightly pressing the inner lower lip (oral mucosa) against the half-spherical lens of the reflection accessory. The duration of the lip measurements was 1 min for averaging 1200 single-sided interferograms providing a spectral resolution of 32 cm^−1^ after Fourier-transformation and phase correction. For examples of single-beam spectra of oral mucosa tissue and gray Spectralon standard with 5% reflectance, see also Figure 3a. The lip spectrum and noise level, obtained from two consecutive tissue measurements with baseline correction, is given in absorbance equivalent units in Figure 3b. The log-converted single-beam lip spectrum is also displayed, which shows differences only in a smooth and constant baseline compared to the –log (reflectance) spectrum. The hardware noise within the log-converted single-beam spectra is smaller by a factor of √2 than that found in the absorbance spectra as calculated from two noise-loaded single-beam measurements.

### 2.2. Calibration Design and Reference Method

The measurements presented here were conducted as part of the initial proof-of-concept study, and although a description of earlier outcomes has been published in previous work [34,35], the dataset is nonetheless well-suited to provide additional insight into various calibration strategies and issues involved. The experiments were in compliance with ethical principles for human studies and required the consent of the informed subject. Spectra of the lip mucosa of a male test person with type 1 diabetes mellitus were recorded under the conditions of a modified oral glucose tolerance test (OGTT) over a two-day trial with parallel measurements of capillary blood glucose concentrations.

The OGTT of the first day started in the morning at a low blood glucose concentration after fasting. For reaching an increased blood glucose level after 90 min, a potion with 50 g of glucose (Dextro OGT by Boehringer Mannheim, Germany) was ingested 20 min after the initial blood glucose testing. Another load of carbohydrates was given so that when the maximum blood glucose concentration was reached, an appropriate dose of insulin was injected to achieve a steady reduction in glucose concentration within another 90 min. The test carried out during the second day started with a slightly larger than above fasting level of blood glucose concentration of the subject. After the lapse of 90 min, the glucose amount of 50 g was taken with some liquid. The total duration for the OGTT experiments was 15 h for collecting our total 133 lip spectra.

Capillary blood glucose reference values were measured using a clinical chemistry-established enzymatic assay (D-glucose, Boehringer Mannheim, Germany) based on both enzymes of hexokinase (HK) and glucose-6-phosphate dehydrogenase (G6PDH), as programmed on the analytical instrument model ACP 5040 (Eppendorf, Hamburg, Germany). The coefficient of variation for the reference measurements was less than 4%, as validated by control sera, which is more accurate compared to many studies using test strip devices with a 15% span of relative uncertainty. Over the two days, capillary blood was sampled with 20 μL capillary pipettes from Brand (Wertheim, Germany) after pricking the fingertip and subsequent recording of the exact sampling times. Blood glucose reference readings obtained during this two-day experiment ranged from 30 mg/dL (1.7 mmol/L) to 600 mg/dL (33.3 mmol/L). The population mean value (c_av_) calculated from these measured values was 301 mg/dL (16.7 mmol/L) with a standard deviation of SD = 168 mg/dL (9.3 mmol/L). As the times of blood sampling for our reference measurements did not match those of the collected spectra (the time gap between two reference measurements was approximately 15 min), interpolation between the capillary blood measurements was carried out by spline approximation for calculating the blood concentration values at the time of the spectral lip measurements.

### 2.3. Optical Data for In Vivo Glucose Calibrations

For elucidating probed tissue volumes and photon penetration depths, the photon fluence rate in turbid media can be estimated by different mathematical tools for calculating the radiative transfer. The basis for such calculations are the tissue-optical properties, i.e., absorption and the scattering coefficients, µ_a_ and µ_s_ (in units of mm^−1^), respectively, and the anisotropy of scattering g (dimensionless). Using the latter two parameters, also the reduced scattering coefficient µ’_s_ = µ_s_ (1 – g) can be calculated. From simple diffusion theory, the optical penetration depth can be estimated as δ = (3 µ_a_ (µ_a_ + µ’_s_)) ^– 1/2^. For more information, the reader is referred to a tutorial on this subject [43]. As evident from the wavelength-dependent optical tissue constants, the average optical pathlength for radiation within mucosa tissue is wavenumber-dependent, as explicated above. Results from Monte Carlo simulations of the radiation transport, simulating the “photon random walk”, were presented in the past for the above-mentioned reflection accessory [41].

**Figure 4:**
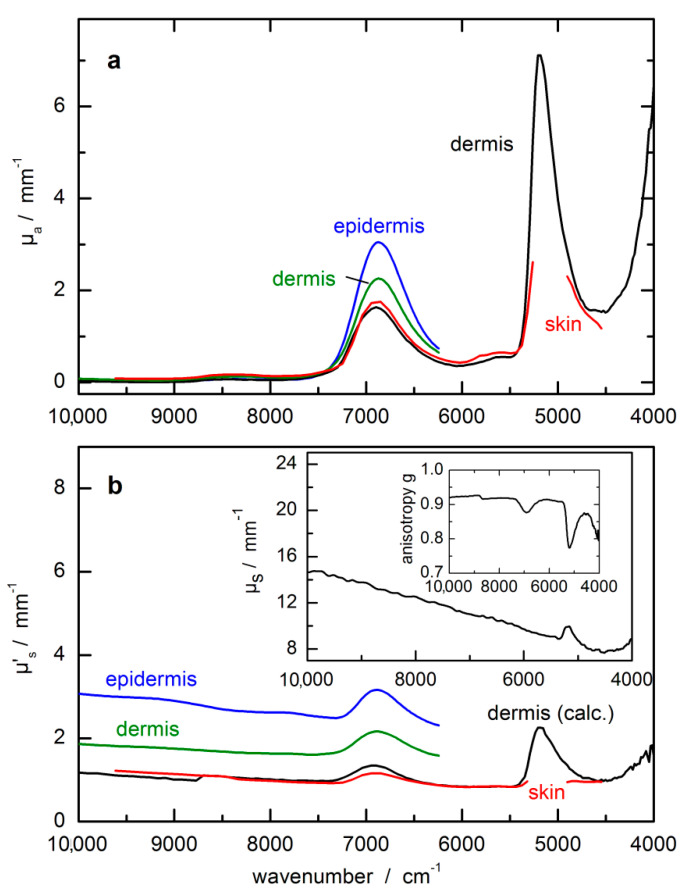
Optical constants of skin, dermis and epidermis from NIR-spectral measurements. (**a**) µ_a_(ῦ) = absorption coefficient; (**b**) µ’_s_(ῦ) = reduced scattering coefficient with µ’_s_(ῦ) = (1 − g(ῦ)) µ_s_(ῦ); this parameter includes the scattering coefficient µ_s_(ῦ) and the anisotropy factor g(ῦ); optical constants were compiled from refs. [44,45,46]; obvious differences in the scattering coefficients within the near-infrared (NIR)-spectral range must be noted.

In Figure 4, optical constants from skin measurements are compiled from three recent publications [44,45,46]. Besides absorption and scattering coefficients, also the anisotropy factor g is shown in the inset of Figure 4b, which is responsible for the main forward scattering characteristics of photons within the NIR spectral range. The optical data for the epidermis and dermis from Salomatina et al. [45] are different from the other compilations, which is certainly understandable for the thin epidermis layer.

**Figure 5:**
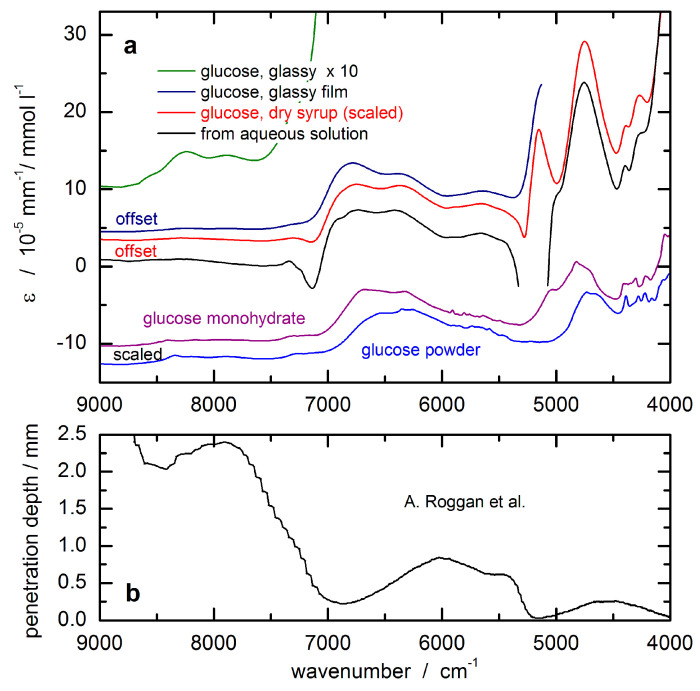
(**a**) Absorptivities of glucose calculated from transmission measurements of aqueous solutions and of glass-like sugars from dehydrated syrup sample (scaled to the aqueous solution phase absorptivities), as well as from glucose powder; (**b**) optical penetration depth based on radiation diffusion theory (from Roggan et al. [46]) used for scaling the glucose response spectrum for the noninvasive assay.

In order to unambiguously detect glucose throughout a complex matrix of several substances within the measured absorption spectrum, a sufficiently large contribution of the analyte signal to the overall spectrum is required that is significantly different from and above the experimentally observed spectral noise. Figure 5 shows the optical constants of glucose obtained from spectral measurements of aqueous solutions in the laboratory. These are of special importance in the field of in vivo NIR spectrometry since it is predominantly the analyte absorption stemming from glucose in the aqueous tissue compartments, i.e., the vascular space and interstitium, that are of interest. The spectra were obtained by scaled subtraction of a pure water absorbance spectrum. The dips in the resulting spectra can be explained by insufficient water compensation attributable to differences in the hydrogen bonding network in pure water and in glucose solutions, respectively. It must be noted that the features of glassy sugar, as-received from syrup preparations after water removal by careful heating, show the same wavenumber dependency as the aqueous solution phase spectrum. Using this technique, the solution-opaque spectral ranges due to large water absorptivities are now accessible, despite still uncompensated water absorption features within the intervals of intense absorption bands. Spectra of crystalline glucose, as well as glucose monohydrate, are also shown, which were scaled for comparison.

## 3. Chemometrics Based on SBC

As the level of familiarity with this method is rather low, a short outline and description of the mathematics is allowed. For the SBC method, the spectral analyte signal is estimated from a physical point of view and the spectral noise by using statistical tools. This can combine the accuracy of an inverse model with relatively low calibration effort and the simplicity of interpretation of a physics-based classical approach. The computational effort of calibration can be considerably diminished with respect to current routine practice using statistical calibrations since the requirement for a large population of calibration samples is no longer necessary. The previously intangible attribute of the analyte’s response specificity is thus based on spectroscopic first-principles, eliminating the need for analytical reference methods for calibration standards, which are essential for PLS calibrations. For computer implementation, the SBC software package was programmed in MATLAB (MathWorks, South Natick, MA, USA).

### Theory and Background

The following NIR spectra are given in units of [AU], from which the analyte concentrations are determined. Here, the analyte of interest is glucose (given in mg/dL). If all spectroscopic factors contributing to the spectrum are available, the experimental NIR spectrum can be described with the following equation:

**x**^T^(t) = y(t) ∙ **g**^T^ + c_1_(t) ∙ **k**_1_^T^ + c_2_(t) ∙ **k**_2_^T^ + … + **i**_baseline_^T^(t) + … + **i**_noise_^T^(t) (1)

where the vector **x**^T^(t) is the experimental spectrum (the transpose sign “T” means that the spectrum is written as a row vector). This vector **x**^T^(t), as well as its compounds, are time-dependent functions of (t). The true, and here sought after, glucose concentration is given as a scalar and described as the “analyte concentration” y(t). The “analyte response spectrum” is **g**^T^ with units of (AU/(mg/dL)); the concentrations (c_1_(t), c_2_(t),… c_n_(t)) and respective response spectra (**k**_1_^T^, **k**_2_^T^,… **k**_n_^T^) contain all information on spectral perturbations that can be explained by tissue components (i.e., water, proteins such as collagen and albumin, blood and interstitial fluid components, and others). The spectra **i**_baseline_^T^(t),…, **i**_noise_^T^(t) include all influencing factors that are produced by the spectrometer and its sampling interface, such as, but not limited to, detector noise, baseline slopes, and shifts, for example, from scattering differences, etc. As the SBC method summarizes all “non-glucose-related” factors in a single expression, Equation (1) can be shortened to:

**x**^T^(t) = y(t) ∙ **g**^T^ + **x**_n_^T^(t) (2)

where **x**_n_^T^(t) represents all factors belonging to the experimental spectrum, such as effects from instrumentation or spectra from interferents, but excludes contributions from the sought-after analyte. The first term, denoted by “y(t) ∙ **g**^T^”, is the “spectral signal”, whereas the second term “**x**_n_^T^” will be noted as “noise”.

The key issue of the SBC method is knowing the response spectrum of the analyte, **g**^T^. However, for a noninvasive glucose measurement from diffuse reflection spectra of skin, the situation is quite different from a transmission measurement with given sample thickness as provided by a cuvette for measuring whole blood or blood plasma as in routine clinical chemistry applications. The noninvasive approach requires not only the glucose absorptivities but also the wavenumber-dependent “effective pathlength” within the probed tissue for a chosen accessory for diffuse-reflection measurements.

The spectral signal and spectral noise can be described by their first- and second-order statistics [37]. The spectral signal can be defined by a mean, $\bar{y}$ ∙ **g**^T^, and a root-mean-square (RMS) term, σ_y_ ∙ **g**^T^, where **g**^T^ is the analyte response spectrum. In the case of people with type-I diabetes, the standard deviation σ_y_ for the varying blood glucose levels y(t) can be as large as around 90 mg/dL. Spectral noise thus can be described by a vector of mean, $\bar{x}$_n_^T^, and a covariance matrix **Σ**, where the latter provides all spectral changes, which occur independently of the sought-after analyte, i.e., the variation from interferents and additional instrumental effects. It is advantageous that for noninvasive glucose measurements, the spectral noise **Σ** can also easily be determined by recording N spectra from healthy people with near-constant glucose concentrations reflecting the spectral tissue variations over time independent of blood glucose changes. By forming these spectra **x**_n_^T^ into a matrix **X**, our covariance matrix **Σ** is calculated as

(3) $$\Sigma \;≅\;{\tilde{X}}^{T}\;\tilde{X}/(N-1)[{AU}^{2}]$$

where the tilde (“~”) indicates a mean-centered matrix. If a “subject-specific” estimate of the spectral noise is required, spectra from a single test subject can be sampled over time to estimate the noise covariance. With regard to the instrument-to-instrument “noise,” the determination of **Σ** virtually always has relatively low experimental effort since reference values of the analyte concentration are not essential, as described above. In addition, should there be any variation in the glucose levels that are present in the experimental spectra collected for estimating **Σ**, the calibration method will still work (for further details, we refer to ref. [33]).

By applying the notation above, the optimal regression vector (also known in literature as “b-vector”) for the analyte determination is calculated by:

**b**_opt(1)_ = **Σ^-^g**/(**g**^T^**Σ^-^ g)** [(mg/dL)/AU] (4)

with **Σ^-^** being the inverse of **Σ**. Please note that Equation (4) provides a mean-square “prediction” error minimum under the condition of unity prediction slope, necessary for measurement purposes, as illustrated by a scatter plot of predicted versus reference concentration values (indicated in the subscript). When **b**_opt(1)_ is applied for the “prediction” of the analyte concentration from a newly measured spectrum, **x**^T^_pred_, its concentration y_pred_ is calculated by:

(5) $$y_{\text{pred}}\;=\;\bar{y}\;+\;{\left(x_{\text{pred}}-\bar{x}\right)}^{T}\;\cdot \;b_{\text{opt}(1)}\;(\text{mg}/\text{dL})$$

with $\bar{y}$ being the mean analyte concentration and ${\bar{x}}^{T}$ being the mean noise spectrum of the individual spectra, which were employed for estimating **Σ**. With these definitions, the RMS prediction error, also known as the standard deviation of (y_pred_ − y), is calculated by:

*SEP*_opt_ = (1**/**(**g**^T^ ∙ **Σ^-^** ∙ **g**))^1/2^ (mg/dL) (6)

If we look into the dependencies of the b-vector optimum (Equation (4)) and the detection limit (Equation (6)), neither are dependent on reference values since these only depend on spectroscopic data.

The issue of selectivity in the multivariate calibration case has often been discussed in the context of performance characteristics of analytical methods. The concept of “net analyte signal”—see, e.g., refs. [27,31,32]—is useful and approximates the correct definition in those stable measurement conditions where, after orthogonalization against all “other” components, there is still a sufficient analyte response spectrum that is well above the instrument noise floor. As noted in a previous publication, the net-analyte concept is insufficient and inconsistent for routine experience in many NIR-spectroscopic and other challenging applications with statistical calibration modeling [47]. The correct definition of selectivity is mathematically straightforward when using the SBC-scheme and nomenclature [33].

A number of important advantages of SBC are evident from the discussion above:

- Laboratory-based analytical work is made virtually needless for calibration for allocating reference values (for validation, this will often still be necessary). Thus, the workload of calibration is significantly lowered when comparing it to statistical calibration effort with PLS;
- The “noise” spectra required for an estimate of the “skin noise” can be sampled from healthy test subjects instead of from people with *diabetes mellitus*. The fact that these “normal subjects” will show only a narrow glucose variance is certainly an SBC advantage;
- Selectivity of response can easily be proven to regulatory agencies and concerned practitioners. Method validation, which requires an application-specific assessment, also becomes easier.

Finally, we remark that the SBC “method” is not an algorithm per se. It uses Equation (4) to compute the b-vector, and for this, the user is asked to provide estimates of both important calibration parameters, i.e., the signal **g** and the noise **Σ**. When both estimates describe reality well, the resulting calibration is the so-called “matched filter” and achieves the globally optimal mean-square error.

## 4. Results and Discussion

Some preliminary information must be mentioned before presenting the results from our two-day OGTT test with a type-1 diabetic subject. For concentration prediction, we used the—log (reflectance) spectra of the inner lip as absorbance equivalent data and an SBC calibration vector calculated as follows: The calibration interval was from 8994–5477 cm^−1^ (115 data points).

First, the spectral noise, **Σ**, was estimated as the sum of four variance terms. As an intrinsic term, the hardware noise can be found on the diagonal matrix elements of **Σ**, which was estimated to be 30 µAU RMS at 6300 cm^−1^. At other wavenumbers, this value was scaled by the inverse of the intensity of the single-beam of the (average) lip spectrum, thus becoming wavenumber-dependent. Second, offset noise—defined as a spectrally flat baseline with an amplitude randomly varying with 50 mAU RMS – was added; third, the spectral variation from the irreproducibility of the lip contact, also known as “lip-noise” covariance, was calculated from a population of differences of spectra less than 8 min apart to minimize residual glucose features. Forth, “water displacement noise” was constructed by using a pathlength scaled water spectrum (see details of the pathlength scaling below), calculated from absorptivity data from Bertie [48], and the amplitude scaled to 2% RMS of displacement. The MATLAB™ codes used in the calculation of these four variance terms were all very similar to the codes given in [33].

The “water displacement noise” was added to the estimate of **Σ** in order to break the unspecific correlation (UC) that exists between the glucose concentration in the skin and its water concentration. The unspecific correlation between the glucose and the water concentrations is due to displacement, i.e., the water concentration is decreasing whenever the glucose concentration is increasing and vice versa. This effect was determined to be the dominant UC effect for the in vitro measurement of glucose [33], and therefore is expected to be important also for the noninvasive case. With the covariance matrix set up in this manner, its inverse was computed at full rank.

The glucose response spectrum used for the SBC calibration was calculated from that of an aqueous solution measured by using a cuvette of 0.5 mm pathlength. It still shows negative features around 7200 cm^−1^ due to water absorbance overcompensation (see Figure 5a). For scaling the glucose absorptivity spectrum, the optical-penetration-depth spectrum, as provided by Roggan et al. [46], see Figure 5b, was used. These values were multiplied by a constant factor of 0.4 in order to account for the reduced glucose concentration in tissue compared to whole blood or blood plasma, by which the spread of experimental blood glucose concentration values was also reproduced.

The experiments with the lip measurements were carried out without an exact repositioning scheme, meaning that the position of the spectroscopically recorded lip area of ca 2 mm diameter was randomly distributed across an area of roughly one cm^2^. Repositioning of the optical probe, for example, during an experiment using a rat animal model (see ref. [32]) led to a significant scatter in glucose prediction, so that the quality of our lip spectral data with regard to reproducibility and low-noise must be highly rated, especially when the second-day spectra are taken into account with the test subject showing more routine in lip measurements. Despite the temperature control of the lip contact area of the accessory, still, temperature gradients can be manifested, as evident from a principal component analysis (PCA) of the spectral population [36]. The dominating factors stem from the water spectrum and its temperature dependency, but also other features arising from methylene stretching overtones of the long-chain acyl groups found in the subcutaneous fatty tissue become visible.

**Figure 6:**
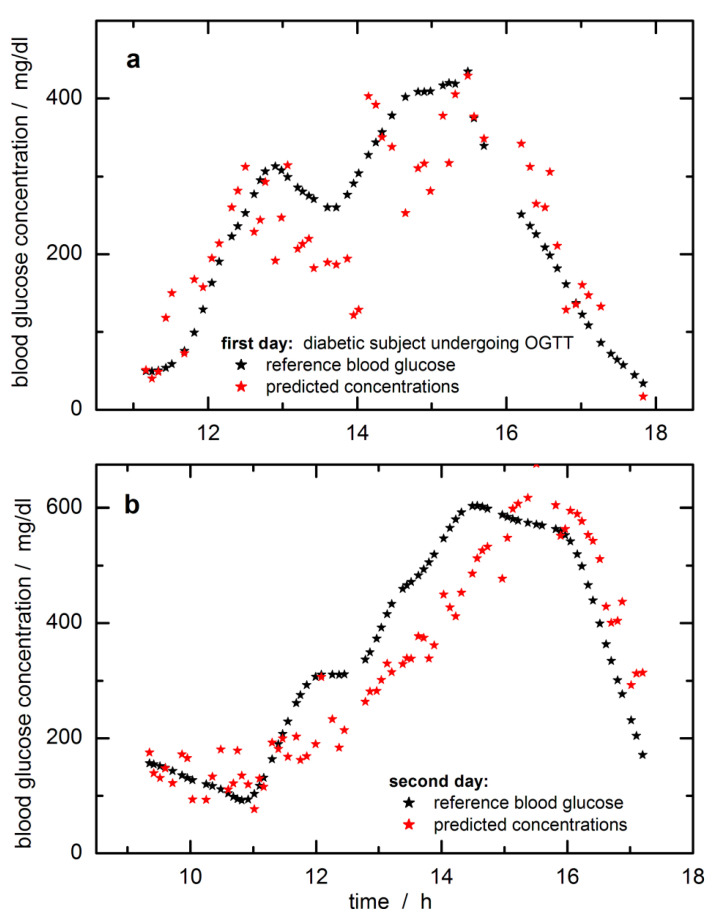
Predictions of tissue glucose concentrations versus blood glucose values for the first day (**a**) and the second day of the modified oral glucose tolerance test (OGTT) (**b**); tissue concentrations were calculated with the SBC-calibration model using reflection lip spectra and glucose absorptivities scaled with photon penetration depth (results were offset corrected, see also text). Spectra and reference blood glucose concentration data are from previous publications [31,33]; calibration interval was from 8994–5477 cm^−1^ (115 data points) with noise covariance calculated from differences of lip spectra less than 8 min apart.

In Figure 6, the time-dependent reference blood glucose concentration values are displayed together with the SBC predictions. The predicted and reference glucose concentration values had been day-wise mean-centered to adjust for a constant offset experienced here. The raw predictions were subtracted by 20 mg/dL to align with the fasting blood glucose level when starting the monitoring over the day. The offset makes sense to “high pass filter out” a noise component that was so far not included in the calibration **Σ.** As a possible explanation, we suppose that the pure water spectrum declared as “water displacement noise” and added to the estimate of **Σ**, was not quite appropriate, since there exist differences for the tissue water due to temperature and hydrogen-bonded molecules of various tissue compounds, which can be sensitively detected within the NIR spectrum. An idea of the complexity of water signatures within lip tissue can be obtained from a principal component analysis (PCA) of the lip spectra, which has been earlier illustrated [36]. At least the first five factors contributing to the spectral variance show features related to water and its dependencies on temperature gradients and differences in the hydrogen-bonded network.

For the first day, during the starting phase, a slightly “running ahead” of the time profile of the tissue glucose is observed compared to the reference capillary concentration values from the fingertip, whereas for other periods, a significant time lag can be noticed. A similar lack of time correlation can be observed for the second day (this trace is also showing a reduced scatter when compared with the prediction data of the first day; note the difference in the *Y*-axis scales). Two days’ worth of data from a single subject is not a large enough data set to allow quantitative statements about “typical” time behavior, but the following conclusions are clear. Relative to the time profile of the blood glucose concentration, the time profile in the skin can lead or lag. In several publications, it has been noticed that a glucose decrease in tissue can drop earlier than found for the vascular compartment, i.e., a glucose decrement in tissue precedes hypoglycemia [49]. On the other hand, also such a feature has been found for situations with a lead-in tissue for glucose increments (see experiment shown in [32]). The lack of time-correlation displayed by the examples in Figure 6 is concerning. The mechanisms causing this discrepancy will need to be studied in the future, and their effects quantified for given segments observed for “typical” patients in typical environments. Especially for skin inserted CGM sensors, the time delay between blood and interstitial glucose profiles has been recently studied several times with two publications given here, one with three different sensors and a second paper dealing with an in silico study [50,51]. In particular, the in silico study is extremely interesting as glucose concentration differences of up to ±40 mg/dL between blood and interstitium were obtained, and time delays up to 25 min were realized. At any rate, even the simplest way of thinking about the glucose-in-the-skin as a time signal leads to a second-order differential equation, i.e., with two inputs, carbohydrate intake making the signal go up and injecting insulin, making it decrease. Which of these two effects wins the race to the measured skin and thus determines the slope (d/dt) of the signal at the time of measurement depends on what the subject did during the previous two hours. Given that people are a bit unpredictable even when performing routines, there cannot be an exactly fixed time-shift relationship to the glucose-in-the-blood. However, we can hope for more-or-less repeatable tendencies of patient behavior.

The SBC method also allows measuring the effect of the hardware noise floor only, i.e., by multiplying it into the b-vector for providing an estimate of the repeatability error, which was calculated to be 35.5 mg/dL RMS. For further comparison, with these data set at hand, several extensive studies have been carried out based on PLS calibrations, and the reader is referred to our publications [33,36]. Best standard errors of prediction could be achieved with variable selection, reaching a SEP = 36.6 mg/dL. Using an impulse invariant designed Butterworth filter of first-order with a time constants of 10 min, the time-dependent blood glucose profiles were shifted for allocating probably concentration reference values more similar to tissue estimates, but SEP improvements of only 2.5 mg/dL could be reached. Arnold and coworkers considered a time shift of 15 min between tissue and blood glucose profile for rat skin [32]. An offset similar as experienced within our SBC study could also be observed when using different daily data sets for calibration modeling; see results illustrated in [33]. Arnold and coworkers investigated the tissue variability and its impact on the PLS regression vectors. Differences in skin inhomogeneity led to vector changes with the effect of offsetting the prediction results.

Since there is no least-squares fitting to blood glucose values (or any other reference values) carried out in SBC calibration, the SBC predictions represent a direct measurement of the glucose concentration in the tissue. Unlike statistical calibrations, PLS, etc., which rely on the correlation between blood glucose reference values and skin spectra, and which are therefore influenced by the dynamic glucose transport processes between vascular and interstitial and intracellular compartments, SBC calibration is not influenced by these processes. It just measures the glucose in the skin. Therefore, it is rather useless to state SEP-values with systematic deviations as illustrated by in silico simulations, which yet considered two compartments only, i.e., vascular and interstitial space. The methodology applied by integral tissue spectroscopy will even show a larger complexity by taking the intracellular compartment additionally into account. To reach similar results with PLS calibrations, a large number of clamp experiments with steady-state conditions would need to be performed to furnish the analyst with the appropriate number of calibration samples and for allowing a comparison based on SEP or similar metrics used for sensor quality assessment such as MARD values (average of the absolute error between all CGM values and matched reference values). A comparison with other vibrational spectroscopy methods [16,17], recently published and mentioned in the beginning, is difficult. Results from the multiperson studies were given as MARD values of 12% for the mid-infrared spectral measurements with photothermal detection, and spectral outliers previously removed [16] or around 24% and larger for noninvasive Raman measurements [17]. For the SBC study, a MARD of 23% can be calculated with the omission of six extreme outlier data.

Since spectroscopy probes the integral tissue glucose whereas reference values used for validation rely on whole blood analysis, diffusion processes within the tissue, especially between the vascular and interstitial compartments leading to a variable temporal shift in both concentration time-series, will certainly need further investigations. It is not clear, unfortunately, whether it will be possible to predict capillary glucose concentration values uniquely from tissue measurements unless other experimental options for avoiding such complications are employed, e.g., the use of pulsatile spectroscopy (plethysmography).

## 5. Conclusions

The validation of calibration models for a noninvasive and transcutaneous blood glucose assay based on NIR spectrometry of skin illustrates a rather critical aspect. For the first time, the so-called “spectral Wiener filter” method, also known as a science-based method of calibration (SBC), was applied to noninvasive glucose measurement. The accuracy was tested on time-sequenced NIR-spectra collected over two OGTT days with better performance on the second day, which allowed conclusions about the behavior of the glucose concentration–time profiles in the blood and in the measured skin volume.

The accuracy achieved is far from that required for a viable noninvasive measurement. Still, the results are useful in a number of ways. First, they demonstrate that SBC calibration is possible also in the noninvasive case, i.e., that workload of calibration can be drastically diminished compared to today’s routine practice with PLS usage. Second, the results indicate that also in the noninvasive case, glucose can be measured in a selective way, i.e., without using unspecific correlation effects like water displacement as a signal, which statistical calibrations have done in the past [35]. However, more work is needed here. The precision of the presented FT-NIR measurement is not good enough, i.e., the prediction scatters error is too large to allow reliable assessment of the magnitude of the prediction slope, or rather: deviation from the ideal value of unity slope. The latter is necessary for an exact, quantitative prove of selectivity of response [39]. The results shown in Figure 6 indicate a slope close to the desired value of one; for further discussion, see also ref. [33]. The accuracy of the scaling applied to transform the aqueous glucose absorptivity spectrum into the response spectrum of the noninvasive case is hard to estimate but is believed to be within several 10% of the true scaling, which is supported by the prediction of the blood glucose concentration range.

The robustness of calibration also must be improved in the future, i.e., additional variance contributions need to be added to the noise estimate **Σ** used in our SBC calibration (with the corresponding potential price in sensitivity) [35]. With in vivo spectroscopy, the variability from physiology, problems with repositioning of skin tissue, temperature gradients, blood flow effects, photon penetration depths, etc., are required to be further studied before calibration robustness for such application can generally be proven (e.g., avoiding systematic errors like the daily offsets experienced in our data set). The SBC prediction results also reveal that a different type of instrument will be more advantageous in the future because FT-NIR spectrometers are influenced by a relatively large hardware (detector) noise in the NIR-spectral range. In summary, the SBC calibration produced in the above way relies entirely on spectroscopy data and knowledge and does not use the laboratory reference values at all. In this regard, it is similar to the established classical calibration methods, but unlike these spectral fitting methods, the SBC result converges against, and therefore in praxis comes much closer to the optimal Wiener filter result.

The fundamental problem to be overcome in the future, however, seems to be the lack of time correlation between the glucose concentrations within the different tissue compartments, i.e., within the vascular, interstitial, and intracellular space. While the relationship of the temporal glucose profiles from the intravascular and the interstitial compartments was studied intensively by various needle-type sensors, the intracellular space also with phosphorylated glucose has so far been paid no attention apart from a few attempts. The effects causing a mismatch between the glucose concentration in blood and integral skin tissue need to be further evaluated. Though the optical spectroscopic methods have always caught much attention, and many different approaches with demanding hardware have been presented during the last 20 years, there seems to be no break-through hovering on the horizon using NIR-spectroscopy of skin tissues. On the other hand, the SBC method can be considered a strong chemometric tool for regression and modeling tasks, and further work into noninvasive testing will be worthwhile.

## Data Availability

The data presented in this study has been described in more detail in previous publications; see refs. [33,34,35,36]; the data presented were reanalyzed with novel, innovative chemometric tools.

## Abbreviations

(AU) absorbance units; (DCCT) diabetes control and complications trial; (MARD) mean absolute relative difference; (NAS) net analyte signal; (NIR) near-infrared; (OGTT) oral glucose tolerance test; (PAT) process analytical technology; (PCA) principal component analysis; (PLS) partial least-squares; (PCR) principal component regression; (RMS) root mean square; (SBC) science-based calibration; (SEP) standard error of prediction, (SMBG) self-monitoring of blood glucose.
